# Intracranial Myeloid Sarcoma Arising Intra-axially From Acute Myeloid Leukemia: A Case Report and Literature Review

**DOI:** 10.7759/cureus.67884

**Published:** 2024-08-27

**Authors:** Akinori Kageyama, Kazuya Motomura, Ayako Motomura, Yasuhiro Nakajima, Takashi Tsujiuchi, Mamoru Matsuo, Sho Akahori, Masaya Watarai, Iori Kojima, Ryuta Saito

**Affiliations:** 1 Department of Neurosurgery, Daido Hospital, Nagoya, JPN; 2 Department of Neurosurgery, Nagoya University Graduate School of Medicine, Nagoya, JPN; 3 Department of Hematology and Chemotherapy, Daido Hospital, Nagoya, JPN; 4 Department of Pathology, Daido Hospital, Nagoya, JPN

**Keywords:** cd34 positive, tumor removal, acute myeloid leukemia, intra-axial tumor, intracranial myeloid sarcoma

## Abstract

Intracranial myeloid sarcoma is a rare brain tumor and an extramedullary manifestation of malignant hematopoietic neoplasms of myeloid origin. A 76-year-old right-handed male patient was initially diagnosed with acute myeloid leukemia (AML; M4Eo). Three years later, the patient experienced headaches, dizziness, nausea, and gait disturbances. Magnetic resonance imaging of the head revealed a mass lesion that appeared to be extra-axial in the right cerebellum with well-defined borders that did not show contrast enhancement and was in contact with the dura mater. The patient underwent surgical tumor resection using the lateral suboccipital approach. The tumor did not attach to the dura mater, indicating susceptibility of an intra-axial tumor. Complete tumor resection was performed. The intraoperative pathological diagnosis revealed the involvement of AML characterized by small round cells diffusely increasing in size with angiogenesis and invasion of macrophages. In conclusion, we present a rare case of intracranial myeloid sarcoma arising intra-axially and originating from an AML that was treated with surgical tumor resection. Although it is difficult to determine whether the tumor was extra-axial or intra-axial on imaging, intracranial myeloid sarcoma should be considered as a differential disease when the patient has a history of hematological neoplasia, such as AML.

## Introduction

Myeloid sarcoma is an extramedullary manifestation of malignant hematopoietic neoplasms of myeloid origin, such as acute myeloid leukemia (AML), chronic myeloid leukemia (CML), and myeloproliferative disorders [1,2]. Intracranial myeloid sarcomas are often continuous with the meningeal or ependymal layers. Rarely, myeloid sarcomas may invade the brain parenchyma and appear as intra-axial masses [1]. On neuroimaging, including head magnetic resonance imaging (MRI), myeloid sarcomas appear similar to meningiomas, metastatic brain tumors, and lymphomas, thereby making the diagnosis solely based on neuroimaging findings challenging [2]. In a large population-based cohort study in Denmark, the prevalence of myeloid sarcoma in 2,261 patients with AML was 9.7%. However, central nervous system involvement due to myeloid sarcoma is rare, occurring in only 0.4% of the patients [3]. Therefore, the true incidence and detailed pathogenesis of intracranial myeloid sarcoma remain unknown because of the paucity of reported cases in the literature.

In this report, we present a rare case of an intracranial myeloid sarcoma arising intra-axially in the posterior fossa, originating from an AML, that was treated with surgical tumor resection.

## Case presentation

A 76-year-old right-handed man was diagnosed with AML (M4Eo) in April 2018. He underwent induction chemotherapy with three cycles of idarubicin and Ara-C, followed by consolidation therapy with three cycles of high-dose Ara-C, and achieved complete remission (CR). After experiencing relapse following the initial remission induction therapy, he was treated with the mitoxantrone, etoposide, and Ara-C regimen as the second-line chemotherapy. Subsequently, upon achieving CR, the disease relapsed twice, and two courses of Ara-C, aclarubicin, and granulocyte colony-stimulating factor (G-CSF) were administered. Unfortunately, by January 2021, the patient was no longer in CR.

**Figure 1:**
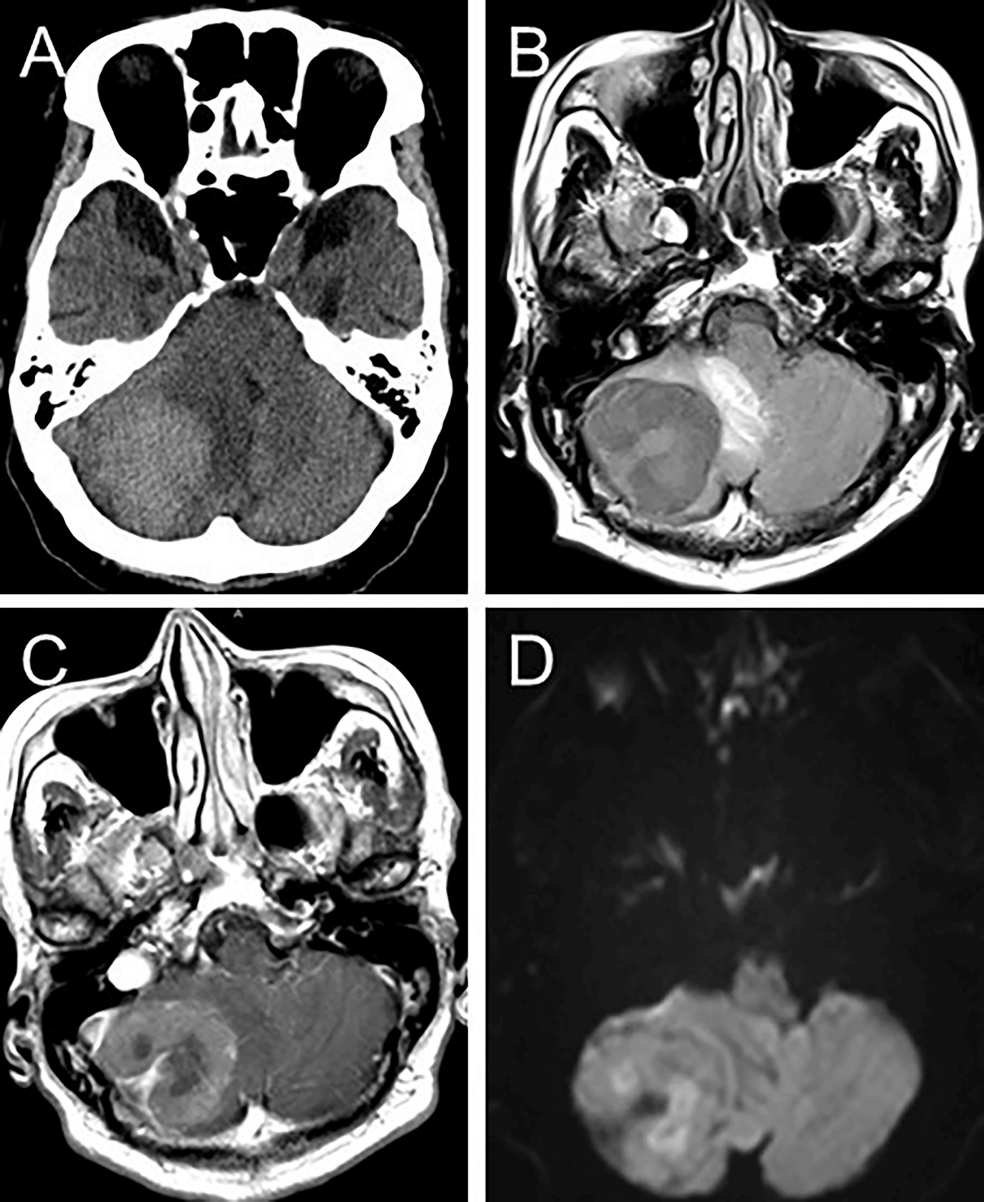
Preoperative CT and MRI (A) Preoperative axial CT reveals a hyperdense mass lesion in the right posterior fossa. (B) Axial T2-weighted MRI reveals a well-defined extra-axial hypointense and (C) T1-weighted MRI with gadolinium enhancement reveals a non-uniform enhancement of the mass in the right posterior fossa. (D) Diffusion-weighted imaging shows an isointensity mass lesion in the right posterior fossa. CT: computed tomography; MRI: magnetic resonance imaging

In February 2021, the patient experienced a headache, dizziness, nausea, and gait disturbance and presented to the emergency department of our hospital. Computed tomography (CT) of the head revealed a hyperdense mass in the right cerebellum, significantly compressing the brainstem and fourth ventricle (Figure 1A). Head MRI revealed an extra-axial mass lesion with well-defined borders that did not exhibit contrast enhancement in the right cerebellum (43×43 mm); it was in contact with the dura mater (Figure 1B, 1C). Diffusion-weighted imaging demonstrated no high-intensity signals (Figure 1D).

**Figure 2:**
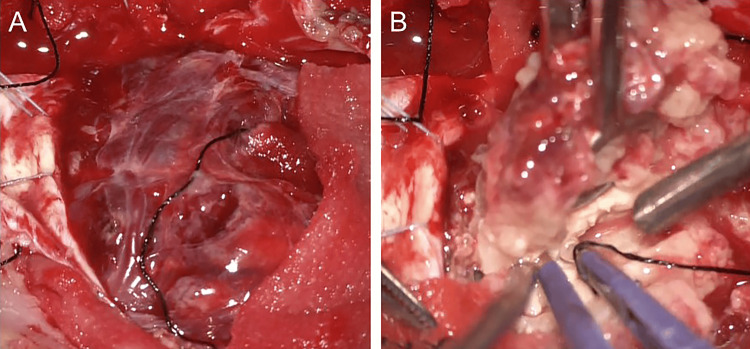
Intraoperative photograph taken after accessing the dura mater (A) The reddish tumor is not adherent to the dura mater. (B) The tumor mass is very hard, with well-defined borders and minimal bleeding.

Six days after the first symptoms, the patient underwent surgical tumor resection with right suboccipital craniotomy in February 2021. After craniotomy, tension in the dura was substantially high because of the large tumor and broad edema. The tumor did not attach to the dura mater, indicating susceptibility to an intra-axial tumor. We performed piecemeal debulking of the tumor. The boundary between the normal brain and the tumor was relatively clear (Figure 2A, 2B).

**Figure 3:**
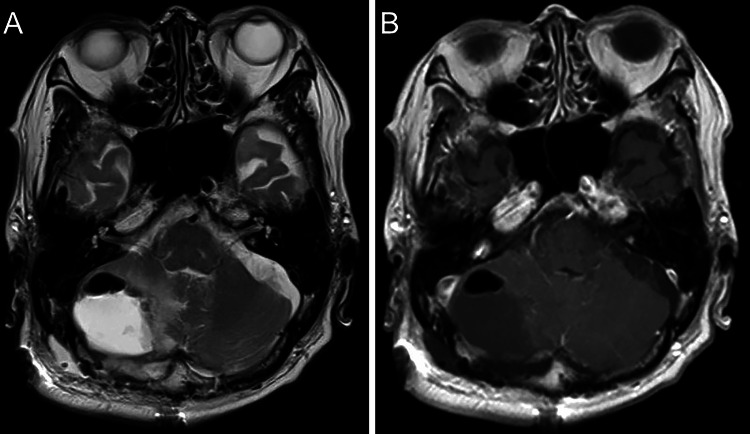
Postoperative MRI (A) Postoperative axial T2- and (B) T1-weighted MRI with (D) gadolinium enhancement reveals no residual tumor in the right posterior fossa. Gross-total removal is achieved. MRI: magnetic resonance imaging

Therefore, a complete resection of the tumor was performed. The intraoperative pathological diagnosis revealed the involvement of AML, characterized by small round cells diffusely increasing in size with angiogenesis and invasion of macrophages. The patient’s general condition and symptoms improved, and postoperative MRI showed no residual tumor (Figure 3A, 3B).

**Figure 4:**
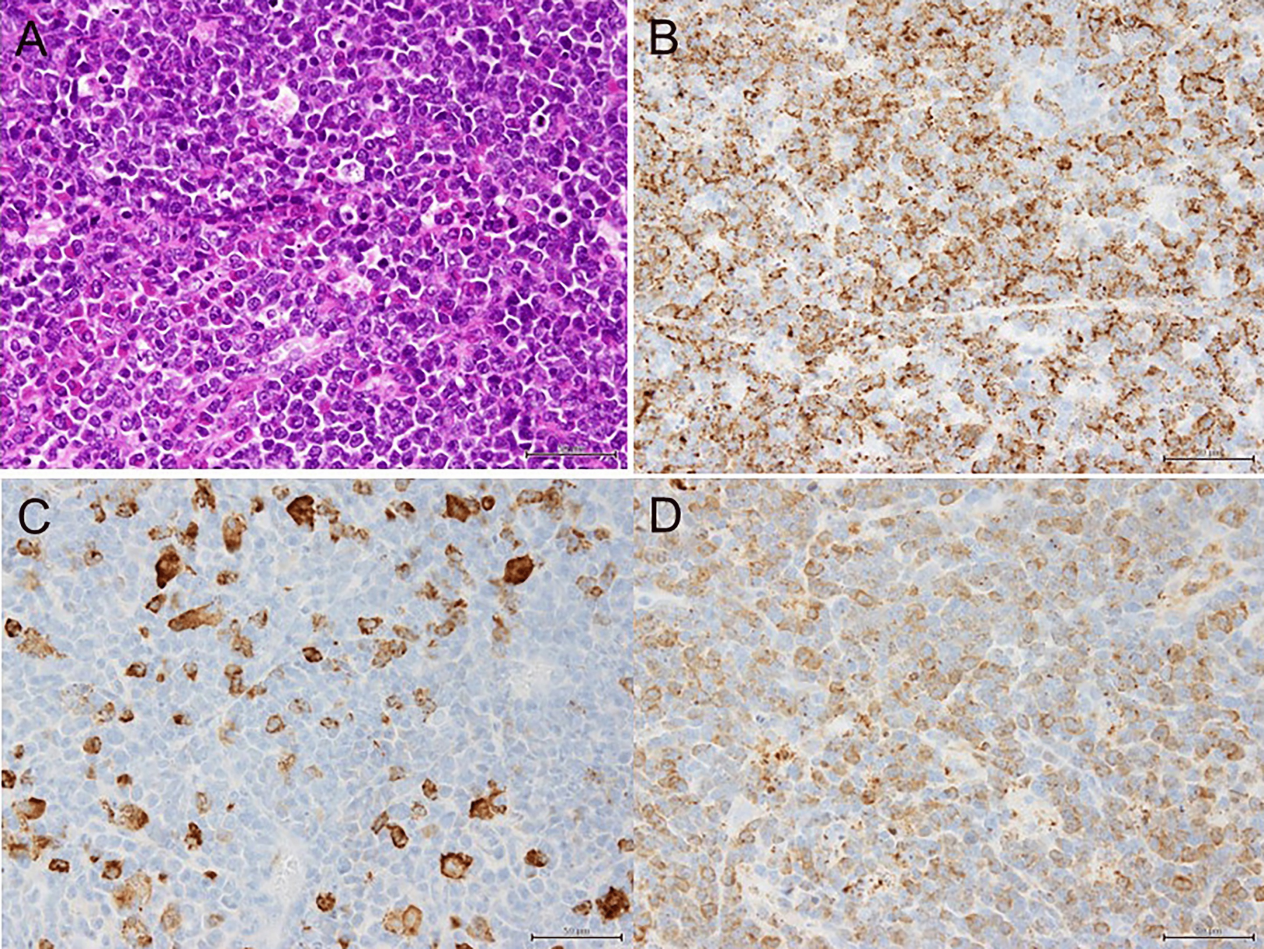
Histopathological findings Histopathological findings reveal a polymorphous infiltrate with numerous immature round cells (A: hematoxylin and eosin stain, ×400), and immunohistochemical staining demonstrates that tumor cells are positive for CD34 (B: ×400), CD163 (C: ×400), and MPO (D: ×400), characteristics of myeloid sarcoma. MPO: myeloperoxidase

The final histopathological examination of the resected tumor revealed sheets of medium-sized immature cells of myeloid origin (Figure 4A). Immunohistochemistry demonstrated cells positive for CD34 (Figure 4B), CD163 (Figure 4C), and myeloperoxidase (Figure 4D), consistent with myeloid sarcoma. The percentage of CD34-positive cells by immunohistochemistry (IHC) was more than 90%. In addition, we performed a peripheral smear, but no blasts were identified. Based on these pathological analyses, the patient was diagnosed with intracranial myeloid sarcoma originating from AML. The patient experienced no postoperative neurological complications and was discharged after 21 days. However, despite the absence of intracranial tumor recurrence, his general condition deteriorated due to worsening AML, and he died seven months after the craniotomy.

## Discussion

Because intracranial myeloid sarcomas are rare, most cases have been documented as case reports. In this context, a systematic review reported that the mean age of patients with intracranial myeloid sarcoma was 34.8 years, 56.6% of patients were male, and 43.4% were female, showing a slightly higher incidence in the male sex [4]. AML is the most common cause of intracranial myeloid sarcoma (68.8%), followed by CML (10.4%). Intracranial sites of occurrence include the temporal lobe (10.3%), cerebellum (10.3%), and falx/parasagittal sinus (10.3%) [4]. Central nervous system involvement of AML does not result in tumor mass formation; it presents as a disseminated disease. In contrast, intracranial myeloid sarcoma is a myeloproliferative disease in which myeloblasts, or immature bone marrow cells, form tumors in the cranium. Intracranial myeloid sarcomas have also been reported in both subtentorial and supratentorial locations [5]. The World Health Organization classification defines intracranial myeloid sarcomas as tumors composed of myeloblasts with or without maturation that arise in anatomical sites other than the bone marrow [6].

The pathogenesis of intracranial myeloid sarcomas remains poorly understood [7]. One theory states that intracranial myeloid sarcoma is caused by leukemic cells in the bone marrow of the skull that infiltrate the subperiosteum and dura mater via the Haversian canal [7,8]. Furthermore, leukemic cells infiltrating the subarachnoid space are thought to cause intracerebral invasion if they disrupt the pia-glial barrier. Therefore, intracranial myeloid sarcoma is often found outside the brain parenchyma but may occasionally be seen as an intracerebral mass.

A few reports investigated whether intracranial myeloid sarcoma caused by AML is intra-axial or extra-axial (Table 1). To date, most reports have described extra-axial-type tumors [9-14]. In one report, a tumor from an AML (M2) extended across the skull bone, intracranially and extracranially (subcutaneously) in the right frontal region, and was clearly an extra-axial type of tumor [9]. In another report, after a 10-year remission of AML, the tumor was located in the medial part of the left temporal lobe, which was attached to the dura mater of the middle cranial fossa and appeared to be a meningioma [14]. In our case, the tumor did not contrast homogeneously, whereas in these reports, the tumor contrasted homogeneously and appeared similar to an extra-axial type of meningioma. Conversely, a 27-year-old male patient with a history of AML was reported to have an intra-axial tumor in the right frontal lobe based on head contrast MRI [13]. In our case, the intracranial myeloid sarcoma was initially difficult to determine whether it was an extra- or intra-axial type of tumor as observed on the head MRI but was confirmed to be an intra-axial type of tumor upon surgical investigation. Therefore, caution should be exercised when interpreting head CT and MRI findings of intracranial myeloid sarcomas. In particular, the possibility of intracranial myeloid sarcomas should be considered because posterior fossa tumors are more likely to be metastatic brain tumors.

**Table 1:** Literature on intracranial myeloid sarcoma originating from myeloid leukemia AML: acute myeloid leukemia; HDAC: histone deacetylase; MIT: mitoxantrone; VP16: etoposide; ACR: aclarubicin

Reference	Age	Sex	Disease status	Previous treatment	Location	Tumor type	Treatment	Outcome
									Nikolic et al. (2003) [5]	45	Male	AML	-	Frontal lobe	Extra-axial	Biopsy. Corticosteroid	Decrease in the size
Nishimura et al. (2004) [9]	30	Female	AML (M2)	Cytarabine+idarubicin+hydrochloride. Cytarabine+etoposide+mitoxantrone+hydrochloride	Frontal lobe	Extra-axial	High-dose cytarabine. Radiotherapy (whole brain 16 Gy, total spinal 24 Gy)	Complete remission									
Hakyemez et al. (2007) [11]	54	Male	AML (M2)	N/A	Temporal lobe	Extra-axial	N/A	N/A
Xu et al. (2009) [12]	34	Female	AML (M2a)	Cytarabine+harringtonine+aclacinomycin	Cerebellopontine	Extra-axial	Craniotomy. Whole brain radiotherapy	Died 11 months after the operation
Akhaddar et al. (2011) [13]	27	Male	AML	None	Frontal lobe	Intra-axial	Daunorubicin+aracytin+imatinib	Complete remission for six months
Lee et al. (2021) [14]	30	Female	AML (M4)	Ara-C+idarubicin. High dose Ara-C	Temporal lobe	Extra-axial	Craniotomy radiation (24 Gy+12 Gy boost). Etoposide+cytarabine+enasidenib	Complete remission for 17 months
The present case	75	Male	AML (M4)	Ara-C+idarubicin. HDAC inhibitor. MIT+Ara-C+VP16. Ara-C+ACR	Posterior fossa	Intra-axial	Craniotomy	Died seven months after the operation

Various studies have discussed the treatment of intracranial myeloid sarcomas [9,12-14]. In our case, due to the deterioration of the patient's general condition caused by worsening AML, only surgical resection was performed, and chemoradiotherapy was not pursued. One report described a patient with intracranial myeloid sarcoma who had sustained CR for six months when treated with chemotherapy (daunorubicin+aracytin+imatinib) alone, without surgery [13]. In another case report, CR was sustained for 17 months after treatment with whole brain irradiation and chemotherapy (etoposide+cytarabine+enasidenib) following tumor resection [14]. If chemoradiotherapy and surgery had been performed in this case, the life expectancy might have been better, as intracranial myeloid sarcoma is radiosensitive, and radiotherapy may improve the prognosis. Moreover, if the lesions are confined to the intracranial space and are completely resected, radiation therapy may result in a favorable prognosis.

A meta-analysis involving 99 patients with intracranial myeloid sarcoma from 82 studies reported that those who received chemotherapy or radiation therapy had a survival advantage over those who did not [4]. Neither surgical treatment nor the extent of tumor resection is associated with mortality [4]. Therefore, we believe that radiation therapy or chemotherapy is recommended because the prognosis of patients with intracranial myeloid sarcoma does not change with the degree of surgical resection. In addition, AML is more advanced in patients with intracranial myeloid sarcoma undergoing local therapy than in those receiving systemic chemotherapy; therefore, AML-type chemotherapy is highly effective. Thus, our patient would have had an improved prognosis if he had been in a better general condition and had received radiation therapy or chemotherapy for AML in addition to surgical tumor resection.

## Conclusions

In this report, we presented a case of an intracranial myeloid sarcoma arising intra-axially from an AML in the posterior cranial fossa. Intracranial myeloid sarcoma often arises from invasion of the periosteum and dura; however, in this case, adhesion was not observed between the dura mater and tumor, suggestive of an intra-axial tumor considering its origin. Even if it is difficult to determine whether the tumor was extra- or intra-axial on several imaging modalities, intracranial myeloid sarcoma should be considered as a differential diagnosis, particularly in patients with a history of hematological neoplasia, such as AML.
